# Electron-Mediated Contrast Mechanisms in Biomedical Imaging: A Narrative Review and the Implication for Emerging Techniques

**DOI:** 10.3390/bioengineering13070831

**Published:** 2026-07-18

**Authors:** Samantha Condo, Reisin Cai, Kejia Cai

**Affiliations:** 1 Department of Biomedical Engineering, University of Illinois at Chicago, Chicago, IL 60607, USA; 2 Blue Valley Northwest High School, Overland Park, KS 66213, USA; rcai@bluevalleyk12.net; 3 Department of Radiology and Biomedical Engineering, University of Illinois at Chicago, Chicago, IL 60607, USA

**Keywords:** electron physics, biomedical imaging, imaging contrast mechanisms, magnetic resonance imaging (MRI), chemical exchange saturation transfer (CEST), electron paramagnetic resonance (EPR), molecular imaging, narrative review

## Abstract

Electron behaviors—including how electrons interact with energy, matter, and magnetic fields—form the foundation of many biomedical imaging modalities, including X-ray imaging, computed tomography (CT), magnetic resonance imaging (MRI), positron emission tomography (PET), optical imaging, electron microscopy, atomic force microscopy, and electron paramagnetic resonance (EPR). These interactions allow visualization of internal structures, molecular processes, tissue composition, oxygenation, redox biology, and high-resolution cellular or surface features. This narrative review provides an overview of key electron-associated mechanisms and their applications in biomedical imaging. Advanced MRI methods, including magnetic resonance spectroscopy, chemical exchange saturation transfer, relayed nuclear Overhauser effect imaging, dynamic nuclear polarization, and hyperpolarized 13C MRI, are highlighted as examples of molecular and metabolic imaging. By comparing modalities across contrast mechanism, spatial and temporal scale, penetration depth, sensitivity, clinical utility, and technological maturity, this review provides a framework for understanding established imaging approaches and contextualizing emerging biomedical imaging technologies.

## 1. Introduction

Medical imaging encompasses a diverse set of technologies that span wide ranges of spatial resolution, contrast mechanisms, and clinical applications. X-ray and computed tomography (CT) visualize anatomical structure through photon attenuation; magnetic resonance imaging (MRI) probes tissue composition and molecular environment through nuclear spin dynamics; positron emission tomography (PET) maps metabolic activity through radiotracer decay and photon detection; and optical, electron, and scanning probe microscopies enable cellular- and molecular-scale interrogation of biological systems. These modalities are often presented as fundamentally distinct, each governed by its own instrumentation, signal source, and clinical use. However, many of their contrast mechanisms share a physical foundation rooted in electronic structure, electron density, excitation, spin dynamics, or interactions involving electron charge and magnetic moment.

Many imaging signals arise from interactions between energy and matter that depend on electron density, binding energy, electronic excitation, or spin-dependent behavior. In X-ray and CT imaging, photon absorption and scattering are determined by electron density, atomic binding energy, and material composition. In MRI, although the detected signal originates from nuclear spins, chemical shielding, molecular motion, dipolar interactions, and susceptibility effects shape resonance frequencies, relaxation rates, and contrast mechanisms. PET relies on positron–electron annihilation to generate detectable photons, while fluorescence imaging arises from electronic excitation and radiative decay within molecular orbitals. Even in modalities that do not directly employ electron beams, such as atomic force microscopy, contrast emerges from interatomic forces shaped by molecular electronic structure.

At smaller spatial and energy scales, specific electronic and spin-dependent mechanisms become more explicit. Scanning electron microscopy exploits elastic and inelastic electron scattering to resolve surface morphology and composition with nanometer-scale resolution. Electron paramagnetic resonance (EPR) directly detects unpaired electron spins to probe redox state, oxygenation, and molecular environment. Dynamic nuclear polarization (DNP) leverages the large magnetic moment of electron spins to enhance nuclear magnetic resonance signals, enabling more sensitive metabolic imaging. Across these techniques, electronic structure and spin dynamics help translate molecular- and quantum-scale processes into measurable imaging signals.

This article is intended as a narrative review and pedagogical synthesis rather than a systematic review of all biomedical imaging technologies. The novelty of the electron-focused framework lies not in proposing new physical laws, but in organizing diverse imaging modalities according to the dominant electron-associated interactions that contribute to contrast generation, signal detection, and translational application. This approach provides a taxonomy for comparing modalities that are often taught or reviewed separately, while preserving important differences in spatial resolution, penetration depth, temporal resolution, sensitivity, cost, instrumentation, and clinical utility.

In this review, we examine major biomedical imaging modalities through this interaction-based framework. We trace how electron density, scattering, excitation, annihilation, spin resonance, shielding, and polarization transfer contribute to image formation in X-ray and CT, PET, optical imaging, SEM, AFM, EPR, and MRI. Particular emphasis is placed on advanced magnetic resonance methods, including DNP, magnetic resonance spectroscopy (MRS), chemical exchange saturation transfer (CEST), and relayed nuclear Overhauser effect (rNOE) imaging, because these methods connect molecular-scale interactions to metabolic and functional imaging. By organizing these techniques within a common physical framework, we aim to provide a coherent foundation for understanding established modalities and for evaluating emerging technologies in biomedical imaging.

## 2. From Electron Physics to Imaging Modalities

For clarity, medical imaging modalities can be organized according to the dominant physical interactions and length scales that determine their contrast mechanisms. Across techniques, image formation often reflects how electronic structure, charge density, or spin dynamics shape the interaction between energy and matter, whether through scattering, excitation, spin polarization, or shielding effects.

Structural imaging primarily exploits electron density, binding energy, and magnetic susceptibility to generate anatomical contrast. In X-ray and CT imaging, photoelectric absorption and Compton scattering depend on the local distribution and binding of electrons, producing contrast between tissues of differing composition. In MRI, although the detected signal arises from nuclear spins, relaxation rates and resonance frequencies are modulated by electron-mediated chemical shielding, dipolar interactions, and molecular motion [1]. At smaller length scales, scanning electron microscopy and atomic force microscopy extend electron-based contrast to cellular and nanometer-scale structures by probing electron scattering and electron-governed interatomic forces, respectively [2,3].

Functional imaging modalities emphasize electron-related energy transfer and spin dynamics to probe physiological and biochemical processes. In positron emission tomography, positron–electron annihilation produces high-energy photons whose coincident detection enables quantitative mapping of metabolic activity. Dynamic nuclear polarization similarly exploits the large magnetic moment of unpaired electrons to transfer polarization to nearby nuclei, dramatically enhancing magnetic resonance sensitivity to metabolic flux [4]. Fluorescence imaging reports on biochemical function through electronic excitation and emission processes that are sensitive to pH, redox state, ion concentration, and molecular binding [5].

At the molecular and cellular level, electronic structure and spin-dependent interactions provide the specificity required for biochemical discrimination. Electronic transitions in fluorophores, electron shielding effects in chemical exchange saturation transfer (CEST) and relayed nuclear Overhauser effect (rNOE) MRI, unpaired electron spins detected by electron paramagnetic resonance spectroscopy, and electron-mediated surface forces measured by atomic force microscopy collectively enable imaging of molecular composition, redox balance, and nanoscale structure [3,6,7]. Table 1 summarizes the practical differences among the modalities discussed in this review. Rather than ranking modalities universally, the table emphasizes how different electron-related interactions lead to distinct tradeoffs in spatial scale, temporal scale, penetration depth, contrast sensitivity, clinical utility, and technical limitations. This interaction-based framework provides the physical context for the modality-specific discussions that follow.

## 3. Scanning Electron Microscopy (SEM) and Atomic Force Microscopy (AFM)

Surface-sensitive microscopy techniques, including scanning electron microscopy (SEM) and atomic force microscopy (AFM), provide high-resolution visualization of tissue, cellular, and subcellular structures. SEM uses a focused electron beam and detects electron- and X-ray-based signals, whereas AFM mechanically senses nanoscale surface forces that arise from interatomic and electronic interactions.

### 3.1. Scanning Electron Microscopy (SEM)

SEM operates by rastering a focused beam of high-energy electrons (typically 1–30 keV) across the specimen surface. As the incident electrons interact with atoms within the sample, a variety of signals are produced, including secondary electrons, backscattered electrons, and characteristic X-rays [2,12].

Inelastic scattering events between incident electrons and surface electrons generate secondary electrons (energies *<* 50 eV), which are highly sensitive to surface topography and form the primary image signal. Since these electrons escape from only a few nanometers beneath the surface, they reveal fine structural details. Elastic collisions between the incident beam and atomic nuclei produce backscattered electrons, whose intensity depends on the atomic number of the specimen, thereby contributing compositional contrast. Characteristic X-rays, arising from inner-shell ionizations followed by electronic transitions, can be analyzed by energy-dispersive X-ray spectroscopy (EDS) to determine elemental composition [23].

Because image formation in SEM depends on electron scattering, parameters such as accelerating voltage, beam current, and working distance determine spatial resolution and depth of field. Modern field-emission SEMs can achieve sub-nanometer resolution under optimized conditions allowing visualization of subcellular and nanoscale structural features [2]. Biological samples, which are inherently non-conductive, must typically be coated with a thin conductive layer (e.g., gold or carbon) to prevent surface charging that would otherwise distort the electron beam and image quality.

### 3.2. Atomic Force Microscopy (AFM)

Atomic Force Microscopy (AFM) is a scanning probe technique that images surfaces by mechanically tracing a sharp nanoscale tip over the specimen. While it does not employ an electron beam, the interactions it measures—van der Waals, electrostatic, and dipole-induced forces—are governed by electron distributions within atoms and molecules [3]. The cantilever tip deflects in response to these forces and a laser beam reflected off the cantilever surface records deflections with sub-nanometer sensitivity.

AFM can operate in several modes. In contact mode, the tip maintains constant interaction with the surface; in tapping mode, it oscillates near resonance frequency, intermittently contacting the sample; and in non-contact mode, it senses attractive forces without direct contact. In conductive AFM or Kelvin probe force microscopy, the technique directly measures electron flow and surface potential to map local conductivity and charge distribution [13].

Unlike electron-based microscopes, AFM can operate in air, vacuum, or liquid environments, making it ideal for imaging biological samples in near-native states. The method achieves vertical (z-axis) resolution below 1 Å and lateral resolution of a few nanometers, enabling visualization of single macromolecules and cellular membrane features [3]. Although AFM detects mechanical rather than radiative signals, its contrast arises from interatomic forces shaped by electronic structure, linking it conceptually to the broader interaction-based framework of this review.

## 4. Optical Imaging

### 4.1. Electron Transitions in Fluorophores

**Figure 1:**
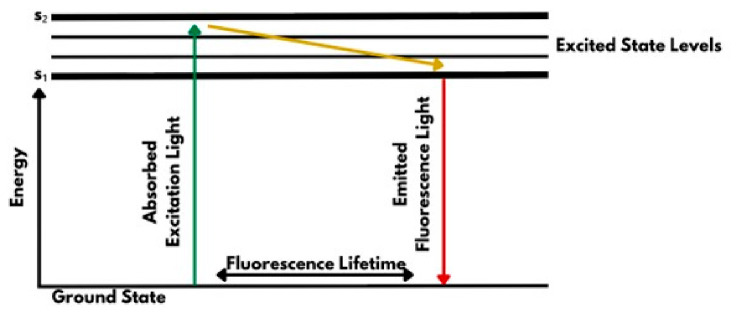
Schematic illustrating fluorescence excitation and emission. The yellow arrow indicates non-radiative relaxation to the lowest excited vibrational level prior to fluorescence emission.

Fluorescence imaging depends on the quantum phenomenon of fluorescence, a type of luminescence in which a molecule emits light after absorbing photons of higher energy. When a fluorophore absorbs excitation light, its electrons are promoted from the ground state (S_0_) to an excited singlet state (S_1_) according to the Franck–Condon principle. Because this excited state is unstable, the electron rapidly relaxes to the lowest vibrational level of S_1_ through non-radiative processes such as internal conversion and vibrational relaxation. The electron then returns to the ground state by emitting a photon of lower energy—a process known as fluorescence emission [5]. These electronic transitions are illustrated schematically in Figure 1.

The difference in energy between absorbed and emitted photons, known as the Stokes shift, arises from energy lost during non-radiative relaxation. This shift is essential for optical separation of excitation and emission light. The efficiency of fluorescence emission is described by the fluorescence quantum yield (Φ*_f_*), defined as the fraction of absorbed photons that are re-emitted as fluorescence. A higher quantum yield indicates a greater probability that an excited electron will relax radiatively rather than non-radiatively, thereby enhancing the observed fluorescence intensity [11].

Fluorescent molecules are typically designed with conjugated *π*-electron systems—alternating single and double bonds—that enable electron delocalization across the molecule. This delocalization lowers the energy gap between the highest occupied molecular orbital (HOMO) and the lowest unoccupied molecular orbital (LUMO), facilitating excitation with lower-energy photons and increasing overall emission efficiency. These electronic properties make conjugated fluorophores, such as rhodamines and cyanines, well suited for imaging applications across a wide spectral range.

### 4.2. Environmental Sensitivity

The fluorescence properties of a molecule are highly sensitive to its electronic environment. Factors such as pH, solvent polarity, metal ion concentration, and protein binding influence both fluorescence intensity and lifetime. Metal ions and proteins can form complexes with fluorophores, altering their electron distribution and transition probabilities, which in turn affect emission wavelength, quantum yield, and stability [24].

pH changes can modulate fluorescence through protonation or deprotonation of functional groups, thereby altering the fluorophore’s electronic configuration. Similarly, quenching processes—such as dynamic quenching by oxygen or static quenching by complex formation—reduce quantum yield by introducing alternative non-radiative pathways. Conversely, fluorophores in rigid, non-polar environments tend to exhibit enhanced quantum yields due to restricted molecular motion and reduced energy loss via vibrational relaxation [11].

These sensitivities make fluorescence imaging uniquely powerful for probing biochemical environments. The electron-level interactions responsible for fluorescence—absorption, excitation, and radiative decay—serve as molecular reporters of pH, ion concentration, and biomolecular binding, linking electronic transitions directly to biological function.

## 5. X-Ray and Computed Tomography (CT)

### 5.1. Electron Interactions in X-Ray Generation

X-ray imaging is driven by high-energy interactions between atomic nuclei and electrons. Inside the X-ray tube, electrons are thermionically emitted from a heated cathode and accelerated across a high-voltage potential toward a dense metal anode, typically tungsten [1]. The high-velocity electrons, upon impact, rapidly decelerate in the Coulomb fields of the anode nuclei. This deceleration produces bremsstrahlung radiation, a continuous spectrum of X-ray photons whose intensity varies with the incident electron energy, tube potential, filtration, and target atomic number [1].

**Figure 2:**
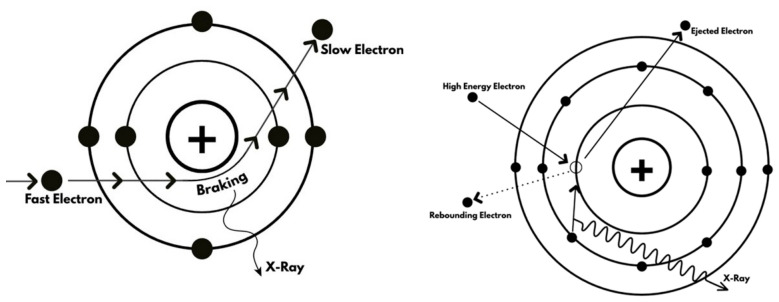
X-ray production mechanisms. (**Left**) Bremsstrahlung radiation generated by electron deceleration in the nuclear Coulomb field. (**Right**) Characteristic X-ray emission resulting from inner-shell electron transitions.

In addition to bremsstrahlung, characteristic X-rays are emitted when incident electrons have enough energy to eject inner-shell electrons, usually from the K or L shells. These vacancies are then filled by electrons from higher-energy shells, and the energy difference is released as photons with discrete energies that are specific to the target material [1]. These emissions reflect electron binding energies, linking the atomic structure of the anode to the spectral profile of the produced X-ray beam. Thus, even before the X-ray beam reaches the patient, electron behavior governs both the generation and energy distribution of the photons used for imaging. These two mechanisms of X-ray production are illustrated in Figure 2.

### 5.2. Tissue Interaction, Attenuation, and CT Contrast

X-rays interact with tissue primarily through photoelectric absorption and Compton scattering in the diagnostic energy range. In the photoelectric effect, an incident photon transfers its energy to a bound inner-shell electron, ejecting the electron and depositing the photon energy locally. This interaction is more likely in materials with higher atomic number and is therefore important for contrast between soft tissue and high-*Z* materials such as cortical bone, iodine, and barium [1]. In Compton scattering, the photon interacts with a more weakly bound outer-shell electron, transfers part of its energy to that electron and is deflected with reduced energy. Compton scattering is strongly related to electron density and is a major contributor to attenuation in soft tissue over much of the diagnostic CT energy range [1,25].

The combined probability of photon absorption and scattering is described by the linear attenuation coefficient, *µ*, which depends on photon energy, physical density, electron density, and atomic composition. For a monoenergetic beam passing through a uniform material of thickness *x*, attenuation follows the Beer–Lambert law:

*I* = *I*_0_*e*^−*µx*^ (1)

where *I*_0_ is the incident X-ray intensity and *I* is the transmitted intensity after passing through the material [1]. For heterogeneous tissue, the attenuation varies spatially along the X-ray path, and the detected signal can be represented as:

(2) $$I\;=\;I_{0}\;exp\left[-\int \mu \left(E,s\right)\;ds\right]$$

where *µ*(*E*,*s*) is the energy- and position-dependent attenuation coefficient along path position *s* [1]. The mass attenuation coefficient, *µ*/*ρ*, is also commonly used to compare attenuation properties independent of physical density, while *Z*_eff_ provides a compact descriptor of the energy-dependent attenuation behavior of mixtures such as biological tissues and contrast agents [1]. CT reconstruction uses projection data derived from the logarithmic attenuation measurement:

(3) $$p=-ln\left(\frac{I}{I_{0}}\right)=\int \mu \left(E,s\right)\;ds$$

By acquiring these projections from multiple angles, CT reconstructs a cross-sectional map of attenuation coefficients that reflects spatial differences in tissue composition.

CT image intensity is commonly expressed in Hounsfield units (*HU*), which normalize tissue attenuation relative to water:

(4) $$HU=1000\times \frac{\mu_{\mathrm{tissue}}-\mu_{\mathrm{water}}}{\mu_{\mathrm{water}}-\mu_{\mathrm{air}}}$$

In this scale, water is approximately 0 *HU* and air is approximately −1000 *HU*, while bone and iodinated contrast agents have positive values due to their greater attenuation. Since attenuation depends on electron density, binding energy, and atomic composition, CT images map spatial variations in photon absorption and scattering across tissue.

For biological tissues and contrast agents composed of mixtures of elements, the effective atomic number, *Z*_eff_, is often used as a practical descriptor of composition. Rather than representing a single elemental atomic number, *Z*_eff_ summarizes the attenuation behavior of a compound or tissue mixture. This concept is especially useful when interpreting contrast from high-*Z* materials, such as iodine- or barium-based contrast agents, and in spectral CT approaches that aim to separate materials based on their energy-dependent attenuation profiles. In the context of the electron-centric framework, *Z*_eff_ reflects how the distribution and binding of electrons within matter shape the probability of X-ray absorption and scattering.

### 5.3. Detector Technologies and Emerging Photon-Counting CT

CT detectors measure transmitted X-rays after tissue attenuation and convert this information into electrical signals for image reconstruction. Conventional energy-integrating detectors rely on scintillator based technologies. In these systems, scintillators absorb X-ray photons and re-emit them as visible light through electron excitation and de-excitation processes; the emitted light is then detected by photodiodes [25]. Thus, the detector stage also depends on electron-mediated interactions, converting X-ray energy into optical and then electrical signals.

Direct-conversion detectors, by contrast, use semiconductors such as cadmium telluride or cadmium zinc telluride to convert absorbed X-ray photons directly into electron–hole pairs, which are collected as electrical signals [8,26]. This direct conversion process forms the basis of photon-counting detector CT, an emerging clinical CT technology in which individual photons can be counted and sorted according to energy thresholds. Compared with conventional energy-integrating CT detectors, photon-counting detectors can reduce electronic noise, improve dose efficiency, increase spatial resolution, enhance iodine signal, and provide multi-energy information from a single acquisition [8]. These capabilities enable improved material decomposition, quantitative imaging, and potential use of lower doses of iodinated contrast material.

Photon-counting CT therefore illustrates how the same physical framework connects tissue attenuation with detector innovation. At the tissue level, contrast arises from photoelectric absorption and Compton scattering. At the detector level, photon energy is converted into charge carriers through semiconductor electron–hole pair generation. Together, these processes illustrate how X-ray and CT imaging translate electron interactions into clinically useful maps of anatomy, tissue composition, and contrast-agent distribution. Thus, CT provides an example in which electron interactions contribute both to tissue attenuation and to detector signal generation.

## 6. Positron Emission Tomography (PET)

Positron Emission Tomography (PET) is a nuclear imaging modality that maps radiotracer distribution to provide functional and metabolic information. Radiotracers labeled with positron-emitting isotopes, such as Fluorine-18 (^18^F), Carbon-11 (^11^C), or Oxygen-15 (^15^O), are administered to the subject. These isotopes undergo *β*^+^ decay, in which a proton in the nucleus is transformed into a neutron, releasing a positron and a neutrino.

**Figure 3:**
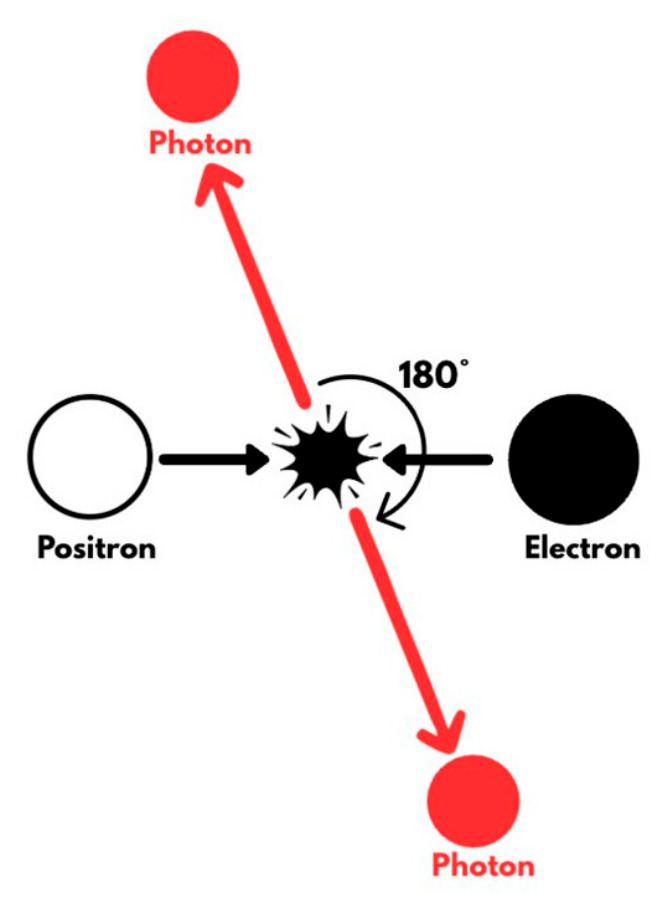
Electron–positron annihilation. When a positron emitted from a radioactive decay encounters an electron, both particles are annihilated, producing two 511 keV photons emitted in opposite directions.

The emitted positron, being the antimatter counterpart of the electron, travels only a few millimeters in tissue before losing kinetic energy through Coulomb interactions with surrounding electrons. Eventually, the positron encounters an electron, and the two particles annihilate, converting their combined rest mass into energy. This process results in the emission of two photons, each with an energy of 511 keV, traveling in nearly opposite directions (180° apart), as governed by the conservation of momentum and quantum electrodynamics [9]. The positron–electron annihilation process underlying PET is illustrated in Figure 3.

The resulting photon pairs are detected by a ring of scintillation detectors surrounding the subject. Each detector element typically consists of a dense scintillator crystal such as lutetium oxyorthosilicate (LSO), lutetium-yttrium oxyorthosilicate (LYSO), or bismuth germanate (BGO), coupled to a photomultiplier tube (PMT) or a silicon photomultiplier (SiPM) [10]. When the 511 keV photons interact with the scintillator via the photoelectric effect or Compton scattering, they generate secondary electrons. These electrons excite the crystal lattice, producing visible photons through de-excitation processes. The emitted light is then converted to an electric signal by the photodetector, allowing for timing and energy measurements critical to coincidence detection and image reconstruction.

Modern PET scanners employ time-of-flight (TOF) detection, which measures the slight difference in arrival times of the two annihilation photons to improve spatial localization. Reconstruction algorithms, such as ordered-subset expectation maximization (OSEM), combine these coincident detection events to generate tomographic maps of tracer distribution [9]. Because the radiotracer’s uptake reflects metabolic or molecular activity, PET provides quantitative functional information about physiological processes such as glucose metabolism, receptor binding, and oxygen consumption.

Overall, PET exemplifies how positron–electron annihilation and scintillator excitation form the physical basis of a nuclear imaging modality that translates subatomic physics into diagnostic molecular imaging. Unlike CT, where electrons shape attenuation probabilities, PET relies on the annihilation of a positron with an electron and the subsequent detection of high-energy photons.

## 7. Electron Paramagnetic Resonance (EPR/ESR)

Electron paramagnetic resonance (EPR), also referred to as electron spin resonance (ESR), is a magnetic resonance technique that detects species containing unpaired electron spins, including free radicals, transition-metal ions, and engineered paramagnetic spin probes [14]. This differs from MRI, which detects nuclear spin transitions, whereas EPR detects transitions of unpaired electron spins whose resonance frequencies, linewidths, and relaxation behavior are highly sensitive to their local chemical and biological environment [14]. Because the electron magnetic moment is much larger than the nuclear magnetic moment, electron spin transitions occur in the microwave frequency range in conventional EPR settings [14]. For in vivo biomedical EPR, however, lower operating frequencies are often used to improve microwave penetration into tissue, creating tradeoffs among sensitivity, penetration depth, spatial resolution, and acquisition speed [27].

When a paramagnetic molecule is placed in an external magnetic field, the unpaired electron spin states undergo Zeeman splitting. Resonance occurs when the microwave photon energy matches the energy separation between spin states:

*hν* = *gµ*_B_*B*_0_ (5)

where *h* is Planck’s constant, *ν* is the microwave frequency, *g* is the electron *g*-factor, *µ*_B_ is the Bohr magneton, and *B*_0_ is the applied magnetic field [14]. In continuous-wave EPR, the magnetic field is most commonly swept at a fixed microwave frequency until this resonance condition is satisfied, although frequency-swept approaches are also possible. In pulsed EPR, short microwave pulses are used to manipulate electron spin coherence and relaxation, enabling measurements of spin dynamics, relaxation behavior, and spatial localization [14]. For EPR imaging, magnetic field gradients can be used to spatially encode the distribution of paramagnetic species, allowing electron spin information to be reconstructed into images.

The *g*-factor provides information about the electronic structure and local molecular environment of the unpaired electron. For a free electron, *g* is approximately 2.0023, but in molecules this value shifts depending on orbital structure, spin–orbit coupling, bonding, and local magnetic fields [14]. EPR spectra are also shaped by hyperfine interactions, which occur when the unpaired electron interacts magnetically with nearby nuclei. Hyperfine splitting can reveal the presence and arrangement of nuclei such as ^1^H, ^14^N, ^15^N, or magnetic nuclei associated with metal centers near the unpaired electron [14]. Thus, EPR does not simply detect the presence of an unpaired electron; when appropriate spin probes are used, it can also report molecular identity, local electronic structure, oxygenation, redox state, and other features of the biological microenvironment.

In biomedical applications, EPR often relies on exogenous spin probes because endogenous free radicals are frequently present at low concentrations, have short lifetimes, or are not sufficiently stable for direct imaging. Nitroxide radicals and hydroxylamine/nitroxide redox couples are commonly used for oxidative-stress and redox-sensitive EPR studies, while trityl or triarylmethyl radicals are commonly used for oxygen-sensitive EPR imaging [15,16,27,28]. Nitroxides are useful because their EPR signal can change as they undergo reduction, oxidation, or conversion reactions in biological systems [27]. Trityl radicals, including OX063- and OX071-type probes, are valuable for oxygen imaging because they can provide narrow linewidths, favorable relaxation properties, and sensitivity to molecular oxygen [15,16,28,29].

One of the most important biomedical applications of EPR imaging is oxygen mapping. Molecular oxygen is paramagnetic and alters the linewidth and/or electron relaxation rates of oxygen-sensitive spin probes through collisional interactions. By calibrating these spectral or relaxation changes against known oxygen concentrations, EPR oxygen imaging can provide quantitative maps of local oxygen partial pressure, *p*O_2_ [15,16]. This capability is especially relevant for cancer, radiation therapy, tissue engineering, wound healing, and cell therapy, where oxygen gradients influence metabolism, hypoxia, viability, and therapeutic response. Recent pulse EPR oxygen imaging studies have demonstrated nondestructive, longitudinal, three-dimensional *p*O_2_ mapping in live-cell and hydrogel systems using trityl OX071-based probes, highlighting the potential of EPR to monitor cellular metabolic activity and engineered biological constructs [29].

EPR also provides a route to redox imaging. Redox-sensitive spin probes can report on reducing or oxidizing environments through changes in signal intensity, spectral shape, relaxation behavior, or probe conversion kinetics. This is particularly relevant for diseases involving oxidative stress, mitochondrial dysfunction, inflammation, ischemia–reperfusion injury, and cancer. Recent advances in low-frequency EPR, rapid-scan EPR, disulfide–dinitroxide spin probes, and image-analysis methods have improved the feasibility of quantitative redox imaging in mammalian tissues, particularly for thiol/glutathione redox status [30].

Despite these strengths, EPR imaging faces important biological and translational limitations. Microwave penetration decreases as operating frequency increases, so in vivo EPR generally requires lower-frequency instrumentation than conventional X-band spectroscopy. However, lower-frequency operation can reduce sensitivity, creating tradeoffs among penetration depth, spatial resolution, acquisition speed, and signal-to-noise ratio [27]. Many endogenous paramagnetic species are too short-lived or too dilute for direct imaging, so exogenous probes must have appropriate stability, biocompatibility, biodistribution, oxygen or redox sensitivity, and clearance [27,28]. Because EPR primarily maps the distribution and spectral behavior of paramagnetic probes rather than detailed tissue anatomy, multimodal co-registration with MRI, CT, or ultrasound is often useful for anatomical interpretation. As a result, EPR is highly valuable for spectroscopy, preclinical imaging, oxygen mapping, and redox biology, but broader clinical translation remains more limited than MRI, CT, or PET.

Within the electron-centric framework of this review, EPR represents one of the most direct biomedical imaging approaches based on electron spin detection. It also provides a conceptual bridge to MRI based technologies such as dynamic nuclear polarization, where the high polarization of electron spins is transferred to nuclear spins to enhance magnetic resonance sensitivity [4,20,31]. In this way, EPR complements metabolic and functional MRI by probing oxygenation, redox biology, and spin-mediated molecular environments that are central to tissue metabolism and disease progression.

## 8. MRI

### 8.1. Electron Roles in Magnet Design

**Figure 4:**
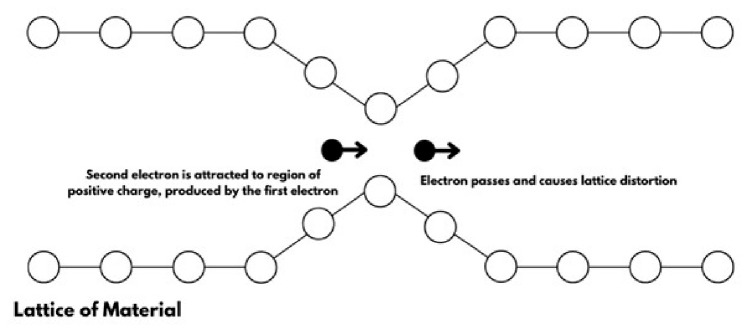
Schematic of Cooper pair formation in a superconducting material. As one electron passes through the lattice, it distorts the positive ion background, creating a region of higher positive charge that attracts a second electron. The resulting bound state (Cooper pair) allows for resistance-free current flow.

MRI differs from EPR in that the detected signal arises from nuclear spins, yet electron physics remains central to both magnet design and molecular contrast mechanisms. MRI depends on strong and stable magnetic fields to align nuclear spins. In high-field systems, the magnetic field is produced using superconducting magnets. The electrical current flows without resistance due to the formation of Cooper pairs. In this quantum state, two electrons with opposite spins and momenta become bound via lattice vibrations (phonons), forming a correlated pair. The electrons move coherently through the superconducting material and are immune to scattering, enabling persistent current loops that generate powerful magnetic fields [1,32]. The formation of Cooper pairs in a conventional superconductor is illustrated schematically in Figure 4.

In low-field or portable systems, permanent magnets are often used. Their magnetic field is generated from aligned electron spins in ferromagnetic materials. The collective orientation of unpaired electron spins within atomic lattices creates a net magnetic field without external power [1].

### 8.2. From Nuclear Spin Signal to Electron-Mediated MRI Contrast

Although MRI magnet design depends strongly on superconductivity and ferromagnetism, the detected signal in conventional MRI arises from nuclear rather than electronic magnetic moments. In most biomedical MRI applications, radiofrequency excitation perturbs the net magnetization of hydrogen nuclei, and the detected signal reflects the precession and relaxation of these nuclear spins in the local magnetic field [1]. Therefore, MRI should not be described as directly imaging electrons in the same manner as electron paramagnetic resonance.

Nevertheless, electronic structure and molecular interactions strongly influence the environments that shape MRI contrast. Local electron shielding alters the effective magnetic field experienced by nuclei and gives rise to chemical shifts. Molecular bonding, motion, dipolar interactions, and magnetic susceptibility contribute to relaxation behavior and local field variations. These effects provide the physical bridge between conventional anatomical MRI and more specialized molecular or metabolic MRI methods, including magnetic resonance spectroscopy, chemical exchange saturation transfer, relayed nuclear Overhauser effect imaging, and dynamic nuclear polarization.

### 8.3. Dynamic Nuclear Polarization (DNP)

Dynamic Nuclear Polarization (DNP) is a technique that enhances nuclear magnetic resonance signals by transferring polarization from unpaired electron spins to nearby nuclei. This enhancement arises as the magnetic moment of the proton is approximately 658 times smaller than that of the electron [33]. As a result, under identical magnetic field and temperature conditions, electron spins exhibit a much higher thermal polarization than nuclear spins. Microwave-driven interactions are used to transfer this electron polarization to nuclear spins, leading to dramatic increases in nuclear spin polarization and corresponding gains in magnetic resonance sensitivity.

Several mechanisms mediate this polarization transfer. The Overhauser effect, predominant in liquids, involves dipolar coupling between electron and nuclear spins that modulates nuclear spin populations via microwave irradiation [31]. In solids, the solid effect, cross effect, and thermal mixing dominate. These rely on simultaneous spin transitions involving one or more electrons and nuclei, depending on the electronic spin density and resonance frequency differences [4]. Collectively, these effects underpin the polarization processes that enable signal enhancement in hyperpolarized MRI probes and contrast agents.

Dissolution DNP extends this electron-to-nuclear polarization transfer into liquid-state NMR and MRI by polarizing a sample in the solid state at low temperature and high magnetic field, followed by rapid dissolution to preserve a nonequilibrium nuclear polarization state [19,20]. This approach can produce signal enhancements exceeding 10^4^-fold over thermal equilibrium, enabling detection of low-concentration nuclei and rapidly evolving metabolic processes that would otherwise be difficult to measure with conventional MRI [19,20].

In preclinical and early clinical studies, dissolution DNP has enabled the development of hyperpolarized ^13^C MRI, allowing real-time metabolic imaging in cancer, cardiac, and neurological applications [20,22]. A common clinical and preclinical example is hyperpolarized [1-^13^C]pyruvate, which enables dynamic observation of downstream metabolic conversion to [1-^13^C]lactate, [1-^13^C]alanine, and ^13^CO_2_/H^13^CO_3^−^_. These measurements provide information about metabolic flux rather than only static anatomy. Early human studies demonstrated the feasibility of hyperpolarized [1-^13^C]pyruvate MRI in prostate cancer, supporting its translational potential for metabolic imaging [21]. Within the electron-centric framework, DNP is therefore one of the clearest examples of how electron spin polarization can be harnessed to amplify nuclear magnetic resonance signals and extend MRI toward molecular and metabolic imaging.

### 8.4. Magnetic Resonance Spectroscopy (MRS), CEST, and rNOE

**Figure 5:**
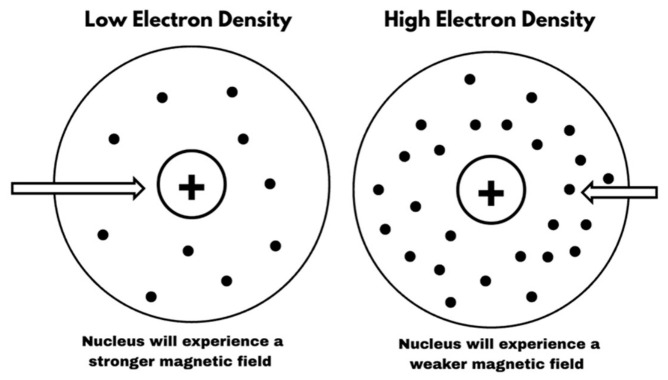
Chemical shift originates from electron shielding. Local electron density induces magnetic shielding that reduces the effective field at a nucleus, shifting resonance upfield; reduced shielding produces downfield shifts. This shielding-dependent frequency separation is the basis of in vivo MRS metabolite identification.

Magnetic Resonance Spectroscopy (MRS) directly probes the chemical environment of nuclei by resolving frequency shifts arising from electron shielding effects. Local electron density and bonding geometry generate induced magnetic fields that partially oppose the applied magnetic field, altering the effective field experienced by a nucleus and producing characteristic chemical shifts. These electron mediated shielding and deshielding effects enable identification and quantification of metabolites such as lactate, choline, creatine, and lipids in vivo, providing a direct link between electronic structure and biochemical composition [1]. The relationship between electron shielding and chemical shift is illustrated schematically in Figure 5. In the context of metabolic MRI, this principle extends beyond conventional anatomical contrast: ^1^H MRS can detect metabolite resonances associated with tissue metabolism, while ^31^P MRS can probe high-energy phosphate metabolism through resonances associated with phosphocreatine, ATP, inorganic phosphate, intracellular pH, and membrane phospholipid metabolism [17].

Chemical Exchange Saturation Transfer (CEST) and relayed Nuclear Overhauser Effect (rNOE) imaging extend these principles by enabling indirect detection of low-concentration molecular pools through the abundant water proton signal. In CEST, selective radiofrequency saturation of exchangeable protons—such as amide, amine, or hydroxyl protons—leads to transfer of saturation to bulk water through chemical exchange. The resonance frequencies, exchange rates, and relaxation pathways governing this process are influenced by local electron distributions, hydrogen bonding, and molecular microenvironment, conferring sensitivity to pH, macromolecular content, and tissue composition [6,7].

In contrast, rNOE contrast arises primarily from dipolar cross-relaxation and saturation-transfer pathways involving non-exchangeable carbon-bound protons in mobile macromolecules, with signal ultimately detected through the water pool rather than through direct observation of the low-concentration molecular species. These interactions depend on molecular motion, spatial proximity, macromolecular structure, and molecular binding, allowing rNOE imaging to probe macromolecular organization, molecular binding, and other features of mobile biomolecular environments [18].

While MRS provides direct spectral resolution of metabolite resonances, CEST and rNOE sacrifice spectral specificity in favor of enhanced spatial mapping and sensitivity. However, CEST and rNOE signals can overlap with direct water saturation, semisolid magnetization transfer, and other exchanging or cross-relaxing pools, so quantitative interpretation requires careful acquisition design, correction strategies, and modeling [6,7,18]. Together, these techniques demonstrate how chemical shielding, exchange dynamics, and dipolar interactions can be harnessed to interrogate tissue microenvironment and molecular composition, reinforcing the central role of molecular-scale interactions in advanced MRI contrast mechanisms.

## 9. Conclusions

Biomedical imaging modalities differ widely in instrumentation, spatial scale, biological specificity, and clinical use, but many of their contrast mechanisms can be understood through interactions involving electron density, electronic excitation, spin behavior, shielding, or charge generation. X-ray and CT depend on photon attenuation governed by electron density, binding energy, and atomic composition; PET relies on positron–electron annihilation and scintillator excitation; fluorescence imaging reflects electronic excitation and emission; SEM and AFM probe electron scattering or electronically shaped surface forces; EPR directly detects unpaired electron spins; and MRI methods are shaped by chemical shielding, molecular motion, susceptibility, dipolar interactions, and electron–nuclear polarization transfer.

The value of this electron-focused perspective is primarily pedagogical and organizational. It does not replace modality-specific physics, nor does it imply that all imaging technologies operate through the same mechanism. Rather, it provides a framework for comparing how different electronic, molecular, and spin-dependent interactions are translated into structural, functional, molecular, or metabolic contrast. This framework is particularly useful for advanced MRI methods such as MRS, CEST, rNOE, DNP, and hyperpolarized ^13^C MRI, where molecular-scale interactions can be used to probe tissue composition, pH-sensitive exchange, macromolecular organization, redox biology, oxygenation, and metabolic flux.

The maturity of these technologies varies substantially. X-ray/CT, PET, fluorescence microscopy, and conventional MRI are established biomedical and clinical imaging tools. Photon-counting CT, advanced CEST and rNOE MRI, hyperpolarized ^13^C MRI, and EPR-based oxygen or redox imaging represent active translational or preclinical areas with expanding biomedical applications. More speculative directions, including quantum sensing, electron-coherence-based imaging concepts, and entanglement-enhanced approaches, remain earlier-stage and require further experimental validation before their clinical feasibility can be assessed. Distinguishing between established, translational, and exploratory technologies is essential for avoiding overstatement while still recognizing how advances in detector materials, molecular probe design, spin physics, and magnetic resonance methods may continue to expand the sensitivity, specificity, and metabolic relevance of biomedical imaging.

## Figures and Tables

**Table 1:** Representative physical and biomedical tradeoffs among imaging modalities discussed in this review. Spatial and temporal values are approximate order-of-magnitude ranges and vary with hardware, acquisition parameters, sample type, reconstruction method, and application.

Modality	Electron related Basis	Spatial Scale	Penetration/Access	Temporal Scale	Sensitivity/Contrast Readout	Clinical Utility and Main Limitation
X-ray/CT [1]	Photoelectric absorption; Compton scattering	~0.5–1 mm clinically	Whole body	Seconds	Attenuation coefficient, HU, electron density, high-*Z* contrast	Routine clinical anatomy; limited soft tissue contrast and ionizing radiation
Photon counting CT [8]	Direct photon to-charge conversion; energy resolved attenuation	Sub-mm clinical imaging	Whole body	Seconds	Energy-bin attenuation, material decomposition, iodine signal	Emerging clinical spectral CT; cost and availability remain limitations
PET [9,10]	Positron–electron annihilation; scintillator excitation	~4–6 mm clinically	Whole body	Minutes	Radiotracer concentration and molecular target uptake	Clinical molecular imaging; ionizing radiation, tracer logistics, lower spatial resolution
Optical fluorescence [5,11]	Electronic excitation and emission	Sub-micron microscopy; coarser for diffuse imaging	Surface to mm–cm depending on wavelength and geometry	ms–min	Fluorescence intensity, lifetime, probe binding, environmental sensitivity	Microscopy, probes, image-guided applications; limited depth and autofluorescence
SEM [2,12]	Elastic/inelastic electron scattering	nm to sub-nm under optimized conditions	Surface/near-surface	Seconds–minutes	Secondary electrons, backscattered electrons, characteristic X-rays	Ex vivo ultrastructure; sample preparation, vacuum, and no routine in vivo imaging
AFM [3,13]	Electron governed surface forces	nm lateral; angstrom-scale vertical	Surface only	Minutes–hours	Cantilever deflection, topography, stiffness, surface forces	Cells, membranes, biomaterials; slow throughput and small field of view
EPR/EPRI [14,15,16]	Unpaired electron spin resonance	Typically mm-scale in vivo; sample dependent	Limited by microwave penetration and resonator geometry	Minutes or longer	Spin-probe distribution, linewidth, relaxation, *p*O_2_, redox state	Preclinical oxygen/redox imaging; sensitivity, penetration, and probe constraints
MRI/MRS [1,17]	Nuclear spin signal shaped by shielding, relaxation, susceptibility	MRI: ∼0.5–2 mm clinically; MRS: coarser/voxel-based	Whole body or organ-level	Minutes	T_1,_ T_2,_ T_2_^*^, susceptibility, chemical shifts, metabolite resonances	Routine clinical soft-tissue imaging; lower sensitivity and longer acquisitions than CT
CEST/rNOE MRI [6,7,18]	Exchange transfer and dipolar cross-relaxation detected through water	Millimeter scale MRI voxels	Organ/regional imaging	Minutes	Water-detected molecular saturation transfer; pH, exchange, macromolecular content	Molecular MRI research/translational imaging; overlapping signals and modeling complexity
DNP/hyperpolarized 13C MRI [19,20,21,22]	Electron to nuclear polarization transfer	mm to cm scale depending on acquisition	Organ/regional metabolic imaging	Seconds–minutes after injection	Transient enhanced 13 C signal and metabolic flux	Early clinical/translational metabolic imaging; polarizer, probe preparation, and rapid decay

## Data Availability

No new data were created or analyzed in this study. Data sharing is not applicable to this article.
